# 14-3-3 Protein Bmh1 triggers short-range compaction of mitotic chromosomes by recruiting sirtuin deacetylase Hst2

**DOI:** 10.1074/jbc.AC120.014758

**Published:** 2020-11-23

**Authors:** Neha Jain, Petra Janning, Heinz Neumann

**Affiliations:** 1 Department of Structural Biochemistry, Max-Planck-Institute of Molecular Physiology, Dortmund, Germany; 2 Department of Chemical Biology, Max-Planck-Institute of Molecular Physiology, Dortmund, Germany; 3 Department of Chemical Engineering and Biotechnology, University of Applied Sciences Darmstadt, Darmstadt, Germany

**Keywords:** genetic code expansion, mass spectrometry (MS), chromatin structure, phosphorylation, histone acetylation, cell cycle, 14-3-3 protein, sirtuin, CIP, calf-intestinal phosphatase, FLuc, firefly luciferase, H3S10ph, phosphorylation of histone H3 serine 10, KDAC, lysine deacetylase

## Abstract

During mitosis, chromosomes are compacted in length by more than 100-fold into rod-shaped forms. In yeast, this process depends on the presence of a centromere, which promotes condensation in *cis* by recruiting mitotic kinases such as Aurora B kinase. This licensing mechanism enables the cell to discriminate chromosomal from noncentromeric DNA and to prohibit the propagation of the latter. Aurora B kinase elicits a cascade of events starting with phosphorylation of histone H3 serine 10 (H3S10ph), which signals the recruitment of lysine deacetylase Hst2 and the removal of lysine 16 acetylation in histone 4. The unmasked histone 4 tails interact with the acidic patch of neighboring nucleosomes to drive short-range compaction of chromatin, but the mechanistic details surrounding the Hst2 activity remain unclear. Using *in vitro* and *in vivo* assays, we demonstrate that the interaction of Hst2 with H3S10ph is mediated by the yeast 14-3-3 protein Bmh1. As a homodimer, Bmh1 binds simultaneously to H3S10ph and the phosphorylated C-terminus of Hst2. Our pull-down experiments with extracts of synchronized cells show that the Hst2–Bmh1 interaction is cell cycle dependent, peaking in the M phase. Furthermore, we show that phosphorylation of C-terminal residues of Hst2, introduced by genetic code expansion, stimulates its deacetylase activity. Hence, the data presented here identify Bmh1 as a key player in the mechanism of licensing of chromosome compaction in mitosis.

In mitosis, cells condense their chromosomes into compact, cylindrical bodies to ensure their faithful mechanical transport during cell division (1). Therefore, ring-shaped protein complexes, the condensins, compact mitotic chromosomes into helical arrays of chromatin loops (2). Condensins are believed to act by loop extrusion (3), fueled by hydrolysis of ATP by their ATPase domains. Loop extrusion has been observed in single-molecule experiments (4, 5) and is supported by structural analysis of DNA-bound condensin subunits (6).

Depletion of condensins has dramatic consequences for mitotic chromosome architecture and mechanical stability (7, 8, 9, 10, 11). However, additional forces and factors must contribute to chromosome condensation because even in the absence of condensins, chromatin still aggregates in mitosis (11, 12). Among the many factors that may contribute to this phenomenon, posttranslational modifications (PTMs) of histones are particularly attractive (13, 14). Histone PTMs may contribute to chromosome condensation in various ways: they could signal the recruitment of effector proteins by serving as recognition marks, might influence the activity of enzymes involved in condensation, or directly control an inherent tendency of chromatin to condense.

The most intensely studied mitotic PTM is phosphorylation of histone H3S10 (15). This hallmark of mitotic chromatin is initially deposited at centromeres by Aurora B kinase as part of the chromosomal passenger complex (16). In budding yeast, phosphorylation of histone H3 serine 10 (H3S10ph) signals the recruitment of lysine deacetylase (KDAC) Hst2 to chromatin in mitosis (17). Hst2 in turn removes acetyl groups from lysine-16 of nearby H4 tails, thereby enabling them to engage with the acidic patch of neighboring nucleosomes. This internucleosomal interaction provides a driving force of chromatin compaction in mitosis, mediating hypercondensation of chromatid arms in late anaphase (17, 18). This short-range compaction acts independently of the axial contraction by condensins (17, 18). Mechanistically, compaction by internucleosomal interactions is initiated by activation of Aurora B kinase at kinetochores. The signal subsequently propagates along chromosome arms in a Shugoshin-dependent process, thereby coupling the ability of chromatin to condense to the presence of a centromere (19). This licensing of condensation by centromeres is a potential mechanism by which yeasts discriminate nonchromosomal DNA from chromosomes, protecting its progeny from infectious genetic material (19).

The spreading mechanism of short-range compaction is essential to create a chromosome-autonomous process. Little is presently known about the molecular mechanism of spreading. Here, we explore how Hst2 recognizes H3S10ph and how this interaction is controlled by the cell cycle.

## Results and discussion

H3 peptides phosphorylated at Ser-10 efficiently recovered Hst2 from yeast whole-cell extracts, demonstrating the significance of H3S10 phosphorylation in recruiting Hst2 to mitotic chromatin (17). However, recombinant Hst2 purified from *Escherichia coli* did not interact with H3S10ph peptides (Fig. S1), indicating that additional factors or PTMs on Hst2 are needed to mediate the interaction.

**Figure 1:**
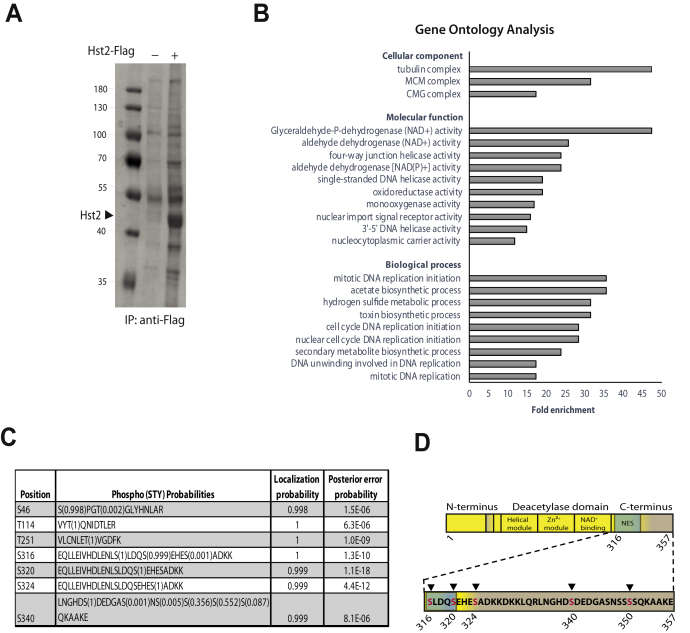
**Identification of Hst2 interaction partners and PTMs by mass-spectrometric analysis**. *A*, immunoprecipitation of Flag-tagged Hst2. Yeast cells expressing Flag-Hst2 were isolated using anti-FLAG M2 beads, analyzed by 10% SDS-PAGE, and stained with InstantBlue. Cells expressing untagged Hst2 served as control. *B*, gene ontology analysis of differentially associated proteins identified in panel *A*. Analysis was performed using the online tool “The Gene Ontology Resource”. *C*, phosphorylation sites identified on Hst2 by MS/MS analysis. For detailed information on HST2 peptide assignments, see Table S4. *D*, the schematic representation of Hst2 C-terminal phosphorylation sites. NES, nuclear export sequence (amino acids 306–317); PTMs, posttranslational modifications.

To identify such factors that recruit Hst2 to the phosphorylated Histone H3 tail, we purified FLAG-tagged Hst2 from yeast extracts (Fig. 1*A*) and analyzed the copurifying proteins by mass spectrometry. We identified several novel interactors of Hst2 with a high fold difference compared with the negative control (untagged Hst2) (Fig. S2 and Table S3). Gene ontology analysis revealed a strong overrepresentation of proteins involved in chromatin related processes, such as replication (Fig. 1*B*). We confirmed the interaction of Hst2 with minichromosome maintenance2, a component of the minichromosome maintenance helicase complex by Western blot (Fig. S3). However, because the association of minichromosome maintenance helicase with chromatin is regulated by other mechanisms than H3 phosphorylation (20), this complex is unlikely to recruit Hst2 to phosphorylated H3 tails.

If the Hst2–H3 interaction requires the presence of PTMs on Hst2, the abundance of the bridging factors in the pull-down might be very low because of the usually low stoichiometry of PTMs. Therefore, we analyzed Hst2 for the presence of phosphorylated residues by MS/MS (Fig. S4) and identified five serine and two threonine phosphorylation sites (Fig. 1*C*). Most sites reside in the unstructured C-terminus of the protein. One of the residues, S340, had been reported previously as being phosphorylated (21). Three serine phosphorylation sites (S316, S320, and S324) cluster downstream of the nuclear export sequence in the C-terminus (Fig. 1*D*) and may therefore be involved in regulating nuclear export.

We hypothesized that phosphorylation of Hst2 is essential for its recruitment to H3S10ph. Typical mediators of interactions between two phosphorylated proteins are 14-3-3 proteins (22, 23, 24). The 14-3-3 proteins are involved in numerous important cellular processes such as transcription (25) and cell-cycle control (26) and are consequently associated with diseases including neurodegenerative disorders and cancer (27).

**Figure 2:**
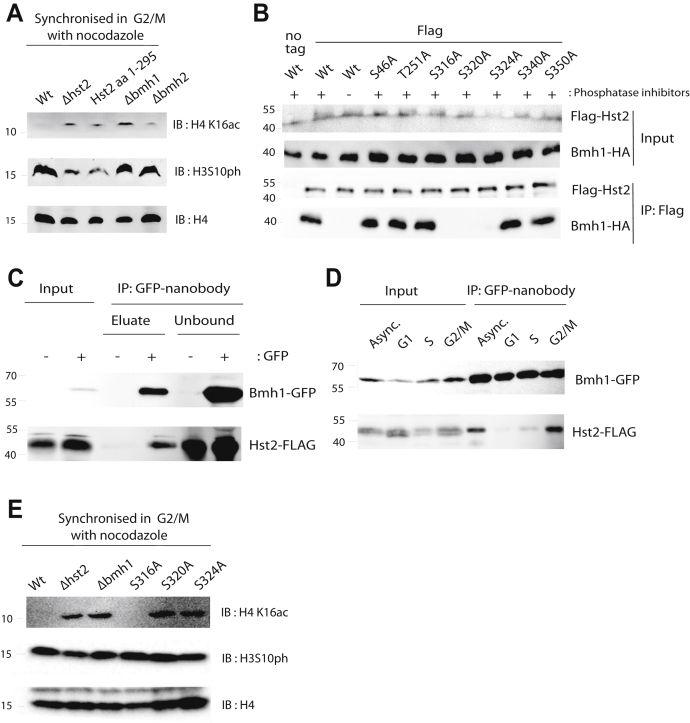
**Interaction of Hst2 with 14-3-3 protein Bmh1 is required for H4K16ac deacetylation in mitosis**. *A*, Bmh1 and the C-terminus of Hst2 are essential for H4K16ac deacetylation in mitosis. Yeast cells were arrested in the M phase with nocodazole, and whole-cell lysates were analyzed by the Western blot with the indicated antibodies. *B*, interaction of Bmh1 with Hst2 *in vivo* depends on phosphorylation of Hst2 S320 or S324. Flag-Hst2 proteins were immunopurified from overexpressing yeast cells and proteins analyzed by Western blot with the indicated antibodies. *C*, Bmh1 interacts with Hst2 *in vivo* at endogenous levels. Bmh1-GFP was isolated from yeast cells with or without genomically Flag-tagged Hst2 using GFP-nanobody beads. Bound proteins were eluted with SUMO protease (releasing the nanobody) and analyzed by Western blot. *D*, Bmh1 interacts with Hst2 *in vivo* at endogenous levels only in the G2/M phase of mitosis. Yeast cells with genomically Flag-tagged Hst2 and GFP-tagged Bmh1 either asynchronous or arrested with α-factor (G1), hydroxyurea (S phase), or nocodazole (G2/M), respectively, were lysed and Bmh1-GFP isolated and bound protein analyzed as shown in panel *C*. *E*, Hst2 S320 and S324 are essential for H4K16ac deacetylation in mitosis. WT and mutant yeast cells were arrested in the M phase with nocodazole and whole-cell lysates analyzed by Western blot with the indicated antibodies. See Figs. S5–S7 for uncropped images.

The interaction of 14-3-3 proteins with H3S10ph is well established across different organisms (28, 29, 30). Conversely, several KDACs are known to interact with 14-3-3 proteins in a phosphorylation-dependent manner (31). For example, SirT2 interacts with 14-3-3β/γ in humans (32) and the homologous Sir2.1 and PAR-5/FTT-2 interact in nematodes (33, 34). Therefore, we tested whether deletion of the budding yeast 14-3-3 proteins, Bmh1 and Bmh2, interferes with deacetylation of H4K16 by Hst2 in mitosis, an established hallmark of short-range chromatin compaction (17). Yeast cells lacking BMH1 that were metaphase-blocked with nocodazole indeed showed elevated H4K16ac levels similar to cells without HST2 (Fig. 2*A*). Deletion of BMH2 had the same effect to a lesser extent, suggesting that both isoforms contribute to the recruitment of Hst2. Because Bmh1 is more abundant than Bmh2 (35), deletion of BMH1 is expected to cause a more severe defect. Truncating Hst2 after residue 295 was sufficient to prevent H4K16 deacetylation, supporting our hypothesis that the unstructured C-terminus of Hst2 (which contains most of the phosphorylation sites) is necessary for its recruitment to H3S10ph.

Next, we tested whether Bmh1 and Hst2 interact physically. Indeed, when we immune-precipitated Hst2 (overexpressed with N-terminal FLAG-tag), Bmh1 efficiently copurified only in the presence of phosphatase inhibitors, indicating a phosphorylation-dependent interaction between both proteins (Fig. 2*B*). The interaction further depended on the presence of serine residues 320 and 324, which we had shown to be phosphorylated in Hst2 (Fig. 1*C*). Mutating the other phosphorylation sites did not affect the interaction with Bmh1. Interestingly, mutation of either Ser-320 or Ser-324 to alanine alone was sufficient to completely abolish Bmh1 binding. Because optimal sequence motifs for 14-3-3 proteins are R-X_2-3_-(pS/pT)-X-P (36), we consider it unlikely that Bmh1 requires the presence of both phosphorylations for binding. It seems more likely that the two phosphorylation sites interact functionally, for example, that phosphorylation of one site depends on the presence of a serine residue at the other site.

Because overexpression of Hst2 might artificially induce the interaction with Bmh1, we repeated the pull-down experiments at endogenous protein levels. Therefore, we precipitated Bmh1-GFP from yeast lysates with genomically FLAG-tagged Hst2 (Fig. 2*C*). Hst2-FLAG specifically co-eluted with Bmh1 depending on the GFP-tag on the latter, confirming that the Hst2–Bmh1 interaction also occurs at endogenous protein concentrations.

Next, we asked whether the Hst2–Bmh1 interaction is influenced by the cell-cycle stage. Therefore, we repeated the pull-down experiments at endogenous protein levels with cell cycle–synchronized yeast cultures (Fig. 2*D*). Pull-down experiments with lysates from yeasts blocked in the G1 phase with α-factor or the S phase with hydroxyurea showed very little interaction of Hst2 with Bmh1. In contrast, we observed efficient interaction of Hst2 with Bmh1 when cells were blocked in the metaphase with nocodazole. This result agrees with our model that Bmh1 recruits Hst2 to chromatin in mitosis when it is needed for short-range chromosome compaction.

Finally, we tested the requirement of individual phosphosites in Hst2 for H4K16 deacetylation in mitosis. Therefore, we created point mutants of Hst2 in which S316, S320, or S324 is replaced with alanine using CRISPR/Cas9. In this case, the S320A and S324A mutants were defective in deacetylating H4K16ac in mitosis, whereas the S316A mutant was functional (Fig. 2*E*). Hence, mutations that interfere with the phosphorylation dependent interaction of Hst2 with Bmh1 also abrogate H4K16ac removal in mitosis.

**Figure 3:**
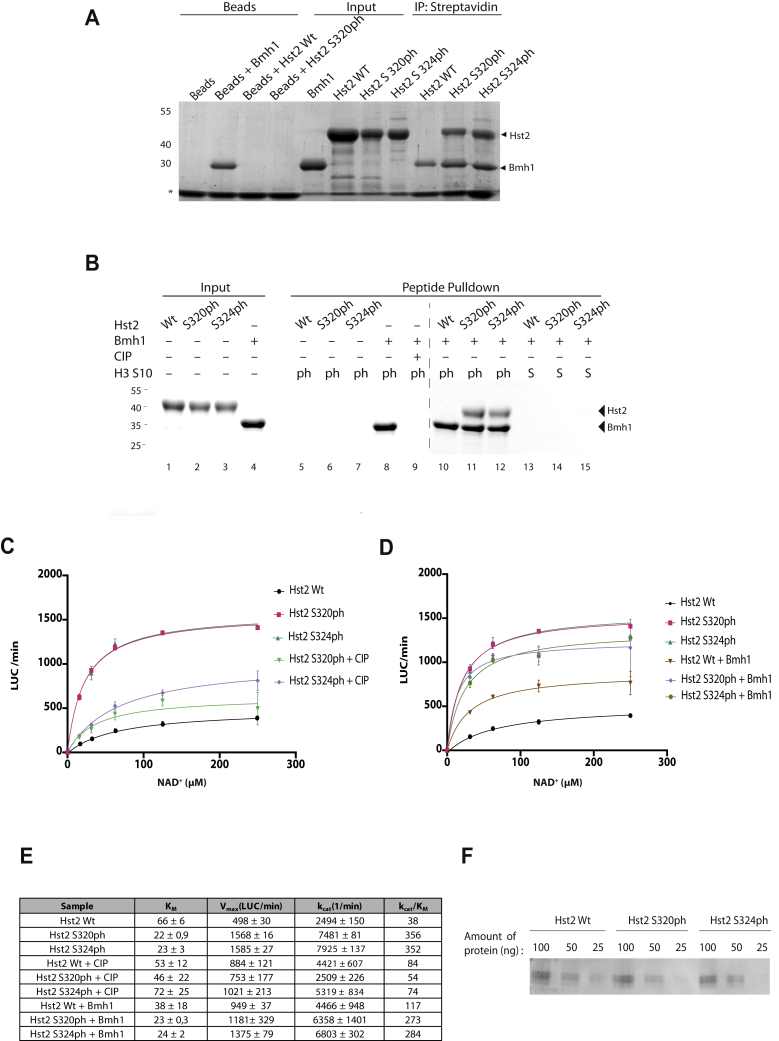
**Phosphorylation of Hst2 enhances catalytic activity and mediates interaction with Bmh1 *in vitro***. *A*, Bmh1 binds to singly phosphorylated Hst2 *in vitro*. Phospho forms of Hst2 produced in *Escherichia coli* were incubated with Strep-tagged Bmh1 on streptavidin beads. Bound proteins were eluted with an SDS sample buffer, analyzed by 10% SDS-PAGE, and stained with InstantBlue. ∗ denotes streptavidin bead–specific band. *B*, Hst2 binding to H3S10ph is mediated by Bmh1 *in vitro*. H3 tail peptides unmodified (S) or phosphorylated (ph) at Ser-10 were immobilized on agarose beads and combined with Hst2 (unmodified, S320ph, or S324ph) and/or Bmh1. Proteins recovered with the beads were analyzed by SDS-PAGE. CIP was used to remove H3S10ph phosphorylation. The image is a composite of two gels run in parallel (indicated by the *dashed line*). *C*, kinetic analysis of Hst2 and the phospho forms S320ph and S324ph. Deacetylation of FLuc K529ac by Hst2 isoforms was measured in a continuous assay format. CIP was used to remove phosphorylation of Hst2. *D*, the impact of Bmh1 on Hst2 activity. Deacetylase activity of Hst2 (with or without phosphorylation) was measured in relation to NAD^+^ concentration in the presence or absence of Bmh1. *E*, kinetic parameters of Hst2 and phospho-Hst2. Error values are calculated using a root mean least squares approach from a double-reciprocal plot. *F*, equal amounts of Hst2 proteins analyzed by 10% SDS-PAGE as loading control for those shown in panels *C*–*D*. CIP, calf-intestinal phosphatase; FLuc, firefly luciferase; H3S10ph, phosphorylation of histone H3 serine 10.

To test the Hst2–Bmh1 interaction under defined conditions, we prepared singly phosphorylated Hst2 proteins in *E. coli* using genetic code expansion (37). Thereby, phosphoserine is encoded in response to amber (UAG) stop codons by an archaeal phosphoseryl-tRNA synthetase–tRNA_CUA_ pair introduced in an *E. coli* strain lacking phosphoserine phosphatase SerB. Suppression of amber codons replacing the codon for phosphorylated serine residues in Hst2 results in the production of site-specifically phosphorylated protein. We produced Hst2 S320ph and Hst2 S324ph and compared their ability to bind to Strep-tagged Bmh1 with unphosphorylated Hst2 (Fig. 3*A*). Both phosphorylated forms of Hst2 were efficiently recovered in these pull-down experiments, whereas unmodified Hst2 did not associate with Bmh1. Hence, both serine residues mutually required for the Hst2–Bmh1 interaction *in vivo* are individually sufficient to mediate the interaction *in vitro*.

Next, we investigated whether Bmh1 is required to bridge the interaction between phosphorylated Hst2 and H3 tails. Therefore, we immobilized H3 peptides with or without phosphorylation on S10 on agarose beads and performed pull-down experiments in the presence of Bmh1 and Hst2 (Fig. 3*B*). Bmh1 bound to the beads in the presence of the phosphorylation on H3 (lane 8) but was unable to bind in the presence of calf-intestinal phosphatase (CIP), which dephosphorylates the peptide (lane 9). Hst2 bound phosphorylated H3 peptides in the presence of Bmh1 when either S320 or S324 was phosphorylated (lanes 11 and 12). In the absence of H3 phosphorylation (lanes 13–15), Bmh1 (lanes 5–7), or Hst2 phosphorylation (lane 10), Hst2 was not recovered with the beads. Hence, Bmh1 is indeed necessary and sufficient to mediate the interaction of phospho-Hst2 with phosphorylated H3 tails.

The phosphorylation of Hst2 may have additional functions in the regulation of Hst2 activity. For example, phosphorylation of S316 could regulate nuclear export of Hst2 because this residue is part of the nuclear export sequence. Furthermore, the C-terminal α-helix of Hst2 has been shown to interfere with NAD^+^ binding (38). Therefore, we measured the activity of recombinant Hst2 with or without phosphorylation of S320 or S324 in dependence of NAD^+^ concentration using acetylated firefly luciferase (FLuc) as substrate (39) (Fig. 3*C*). The phosphorylated forms of Hst2 both showed a 3-fold increase in *V*_max_ and a 3-fold decrease in K_M_ for NAD^+^, resulting in an almost 10-fold higher catalytic efficiency. The enhanced catalytic efficiency was reverted by preincubation of the phosphorylated proteins with CIP, confirming that the presence of the phosphorylation is essential for the effect. The addition of Bmh1 to the deacetylation reaction did not further enhance the activity of phosphorylated Hst2 (Fig. 3*D*). Hence, phosphorylation is sufficient to fully activate Hst2 activity.

This indicates that the unphosphorylated Hst2 C-terminus acts like a mixed noncompetitive inhibitor that is masked by phosphorylation. This can be interpreted in the way that binding of the C-terminus to the catalytic core reduces the affinity for NAD^+^ (increasing K_M_) and also slows catalysis (reducing *V*_max_). The regulation of the catalytic activity by phosphorylation of C-terminal residues appears to be a conserved mechanism that has also been observed for the human Hst2 homologue, SirT2 (40, 41).

## Conclusions

**Figure 4:**
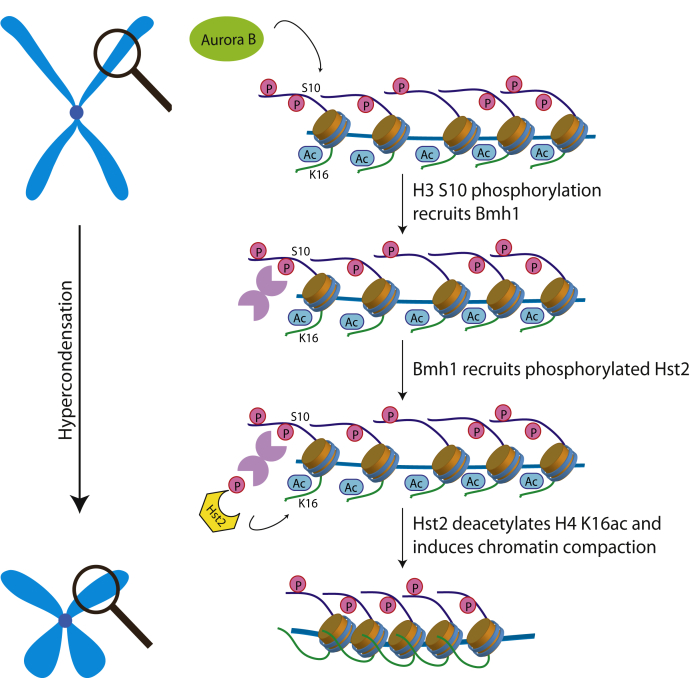
**Bmh1-mediated recruitment of phosphorylated Hst2 to mitotic chromatin induces chromatin compaction.** Phosphorylation of H3 tails by mitotic kinases creates a binding site for 14-3-3 protein Bmh1, which in turn elicits recruitment of Hst2, leading to removal of H4K16ac and chromatin compaction.

Chromosome condensation in mitosis is licensed by kinetochores *via* the recruitment of Shugoshin and Hst2 (19). Phosphorylation of H3S10 by Aurora B kinase is a central event in this process that mediates deacetylation of H4K16ac by Hst2 (17, 42). Here, we demonstrate that binding of Hst2 to this mark is mediated by Bmh1, which simultaneously binds the phosphorylated H3 tail and C-terminally phosphorylated Hst2 (Fig. 4).

Our results show that phosphorylation of Hst2 S320 or S324 is necessary and sufficient to induce the interaction with Bmh1. In contrast, mutation of either site to alanine is sufficient to prevent co-immunoprecipitation of Hst2 and Bmh1 *in vivo*. This may be the result of a complex interplay between these sites *in vivo* such that the phosphorylation of one site depends on the presence of a serine residue at the other site. Alternatively, the *in vitro* pull-down experiments may require less-affine interactions because the interaction partners are present at much higher concentrations in the assay. Interestingly, recruitment of human SirT2 to chromatin is also regulated by phosphorylation (40, 43), suggesting that a similar mechanism might act in mammalian cells.

An important open question is how compaction spreads from kinetochores along chromosome arms. The multivalent nature of the interaction of chromatin, Bmh1-dimers, and Hst2 (which can form trimers *in vitro* (38)) may facilitate liquid-liquid phase separation. This hypothesis is supported by the presence of polyglutamine stretches at the C-terminus of Bmh1 and Bmh2. These motifs are known to interact with nucleic acids and are often involved in phase-separation processes (44).

## Experimental procedures

### Plasmids and strains

Overexpression of Hst2 proteins was from 2-μ plasmids under the control of the HST2 promoter and terminator. For expression in *E. coli*, ORFs of HST2 and BMH1 were cloned in pCDF-DUET with purification tags. Mutations were introduced by QuikChange mutagenesis. Details can be found in Supplementary Materials. Yeast strains were constructed in the S288C background according to standard procedures. The strains used in this study are listed in Table S1.

### Cell-cycle arrests

To synchronize cells in G1, the mating pheromone α-factor (GenScript) was added to log-phase cultures grown to absorbance at 600 nm of 0.4 to 0.6 (MATa yeast strain BY4741) to a final concentration of 15 μg/ml. Cells were incubated for 1 h at 30 °C at which time an additional dose of 7.5 μg/ml α-factor was added and cells incubated for one more hour. Cells were monitored periodically by microscopy. Cells were collected by centrifugation at 4000 rpm for 2 min and processed for further experiments. To synchronize cells in the metaphase, one dose of nocodazole (15 μg/ml, Sigma Aldrich) was added to log-phase cultures (absorbance at 600 nm = 0.5) for 2 h. The cells were monitored by microscopy and collected as above. To synchronize cells in the S phase, 100-mM hydroxyurea (Sigma Aldrich) was added to exponentially growing cells in yeast extract, peptone, dextrose medium (absorbance at 600 nm ≈ 0.3) and incubated for 2 h at 30 °C, after which they were monitored by microscopy and collected as above.

### Protein purifications

#### Hst2

*E. coli* BL21 (DE3) transformed with pCDF-His6-yHst2 were grown overnight at 37 °C in the LB containing 50 μg/ml spectinomycin. Next day, the preculture was used 1:50 to inoculate 4-L LB medium and protein expression induced with 0.5-mM IPTG at absorbance at 600 nm = 0.5, and incubation continued overnight at 15 °C. Cells were harvested, suspended in 20-mM Hepes, pH 7.5, 200-mM NaCl, 20-mM imidazole, 3-mM β-mercaptoethanol, and 1-mM PMSF supplemented with lysozyme (∼0.5 mg/ml), DNase (1 mg), protease inhibitor cocktail (Roche), and disrupted with a pneumatic cell disintegrator. Soluble His-yHst2 was purified by Ni-NTA affinity chromatography using 20-mM Hepes, pH 7.5, 200-mM NaCl, 20-mM imidazole, 3-mM β-mercaptoethanol for binding and washing and additional 200-mM imidazole for elution. The eluate was concentrated and separated by gel filtration in 20-mM Hepes, pH 7.5, 100-mM NaCl, 0.5-mM Tris(2-carboxyethyl)phosphin (TCEP) on a 16/60 Superdex 75 column (GE healthcare, UK). Hst2-containing fractions were pooled, concentrated in a microfiltrator, and stored in aliquots at −80 °C.

#### Bmh1

Protein expression conditions were the same as for Hst2. Cells were lysed in 50-mM Tris, pH 8, 300-mM NaCl, 3-mM β-mercaptoethanol, and 10% glycerol. The supernatant was incubated with Pierce High Capacity Streptavidin Agarose, washed with the same buffer, and Strep-Bmh1 was eluted with additional 10-mM desthiobiotin. Strep-Bmh1 was further purified on a Superdex 200 column (GE Healthcare) in 20-mM Tris, pH 8, 100-mM NaCl, 0.5-mM TCEP, and 10% glycerol.

#### Hst2 S320ph and S324ph

BL21 ΔserB (DE3) cells containing pKW2-EF-Sep (a chloramphenicol-resistant plasmid containing SepRS2, pSer-tRNAB4_CUA_, and EF-Sep (37)) were transformed with pCDF-His-yHst2 S320TAG or S324TAG. Cells were grown at 37 °C in the LB medium containing 50 μg/ml spectinomycin and 34 μg/ml chloramphenicol and used next day to inoculate 4 L LB-SC 1:50. Protein expression was induced at absorbance at 600 nm = 0.5 with 1-mM IPTG, and cells were harvested after 4 h at 37 °C. Purification followed the same protocol as for unmodified Hst2. All proteins were stored at −80 °C.

### Coprecipitation experiments

BY4741 cells were transformed with plasmid pRS423-Flag-Hst2 and grown to absorbance at 600 nm = 1.7 to 3.0. Cells from 1 L were resuspended in PBS supplemented with protease inhibitors (1-mM phenylmethanesulfonyl fluoride and each 5 μg/ml chymostatin, leupeptin, aprotinin, and pepstatin A) and (where indicated) a phosphatase inhibitor cocktail (PhosSTOP Sigma Aldrich). Subsequently, flash-frozen cell nuggets were lysed by milling (RETSCH ZM 200 Ultra Centrifugal Mill), thawed, and centrifuged (20,000 rpm, 4 °C for 15 min). The supernatant (total protein concentration ∼3 mg/ml) was incubated with ANTI-FLAG M2 agarose beads (Sigma Aldrich) at 4 °C for 1 h with agitation. Beads were washed six times with 500-μl PBS containing 0.2% Triton X-100 and finally eluted by boiling in an SDS sample buffer. Proteins were analyzed by SDS-PAGE and the Western blot (antibodies are listed in Table S2).

### *In vitro* pull-downs

Purified, recombinant Bmh1 and Hst2 isoforms (0.3 mg each) were mixed with 20-μl 50:50 slurry of Avidin Agarose Resin (Fisher Scientific) in 80 μl of Bmh1 size exclusion chromatography buffer (20-mM Tris, pH 8, 100-mM NaCl, 0.5-mM TCEP, 10% glycerol), incubated for 1 h at RT with shaking (300 rpm) and subsequently washed three times with PBS. Proteins were eluted by boiling in the SDS-PAGE sample buffer, analyzed by 10% SDS-PAGE, and stained with InstantBlue.

For H3 peptide pull-down reactions, 5-mg peptide was coupled to N-hydroxysuccinimide-activated Sepharose 4B (GE Healthcare Life Sciences). To prepare unphosphorylated H3S10 peptide for control reactions, beads were incubated with CIP at 37 °C for 1 h.

Purified, recombinant Bmh1 and unmodified or phosphorylated Hst2 isoforms (0.3 mg each) were mixed with 20-μl peptide-coupled beads in 80 μl of Bmh1 size exclusion chromatography buffer (20-mM Tris, pH 8, 100-mM NaCl, 0.5-mM TCEP, 10% glycerol), incubated for 1 h at RT with shaking (300 rpm) and subsequently washed three times with PBS. Proteins were eluted by boiling in the SDS-PAGE sample buffer, analyzed by 4 to 15% Criterion TGX Stain-Free Protein Gel (Bio-Rad) and stained with InstantBlue.

### Luciferase-based KDAC assay

The KDAC activity was measured in a continuous assay format using FLuc K529ac (39). Reactions contained 200-nM Hst2 (unmodified or phosphorylated), NAD^+^ at concentrations from 0 to 4 mM, and FLuc K529ac (suitably diluted to match the sensitivity of the luminometer) in 50-μl KDAC buffer (25-mM Tris/HCl, pH 8.0, 137-mM NaCl, 2.7-mM KCl, 1-mM MgCl_2_, 1-mM GSH). To assay luciferase activity, an equal volume of 40-mM tricine, pH 7.8, 200-μM EDTA, 7.4-mM MgSO_4_, 2-mM NaHCO_3_, 34-mM DTT, 0.5-mM ATP, and 0.5-mM luciferin was added and luminescence recorded for 30 min at 30 °C in a FLUOStar Omega Microplate Reader (BMG LABTECH). All experiments were executed in triplicate and averaged, and reactions without the enzyme were used for background subtraction. Initial rates were determined from the linear phase of the reactions. The kinetic parameters (apparent *K*_M_ and *k*_cat_) were obtained by fitting the data to the nonlinear regression Michaelis-Menten model in GraphPad Prism 8 software.

## Data availability

Mass spectrometry raw data and MaxQuant results are available in MassIVE: (https://massive.ucsd.edu/ProteoSAFe/static/massive.jsp; project ID MSV000086283). All other data are included in the article.

## Conflict of interest

H. N. holds a patent on the lysine deacetylase assay used in this manuscript and is the founder of the Steinbeis Research Center Protein Engineering.
