# Supplementary material for: 14-3-3 Protein Bmh1 triggers short-range compaction of mitotic chromosomes by recruiting sirtuin deacetylase Hst2
Source: J Biol Chem. 2020 Nov 23;296:100078. doi: 10.1074/jbc.AC120.014758 (PMC7948448; doi:10.1074/jbc.AC120.014758)
Supplement: Supplementary Figures and Tables [file mmc1.pdf]

14-3-3 protein Bmh1 triggers short-range compaction of mitotic chromosomes by recruiting sirtuin deacetylase Hst2

Neha Jain[1], Petra Janning[2], and Heinz Neumann[1,3]

Supplementary Material

[1] Department of Structural Biochemistry, Max-Planck-Institute of Molecular Physiology, Otto-Hahn-Strasse 11, 44227 Dortmund, Germany

[2] Department of Chemical Biology, Max-Planck-Institute of Molecular Physiology, Otto-Hahn-Strasse 11, 44227 Dortmund, Germany

[3] Department of Chemical Engineering and Biotechnology, University of Applied Sciences Darmstadt, Stephanstrasse 7, 64295 Darmstadt

Correspondence: heinz.neumann@mpi-dortmund.mpg.de

A

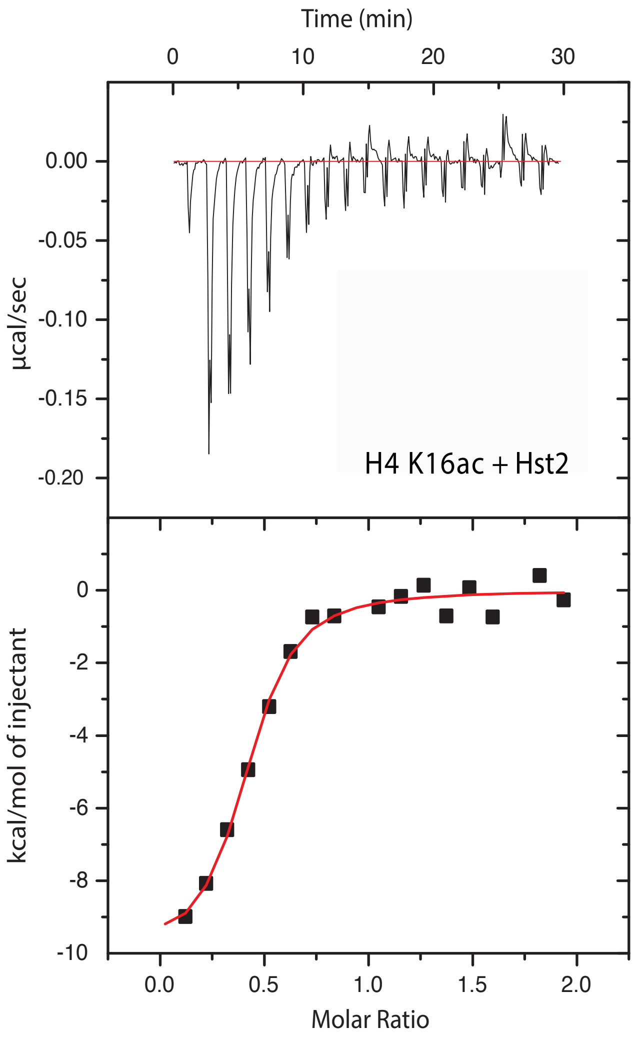

B

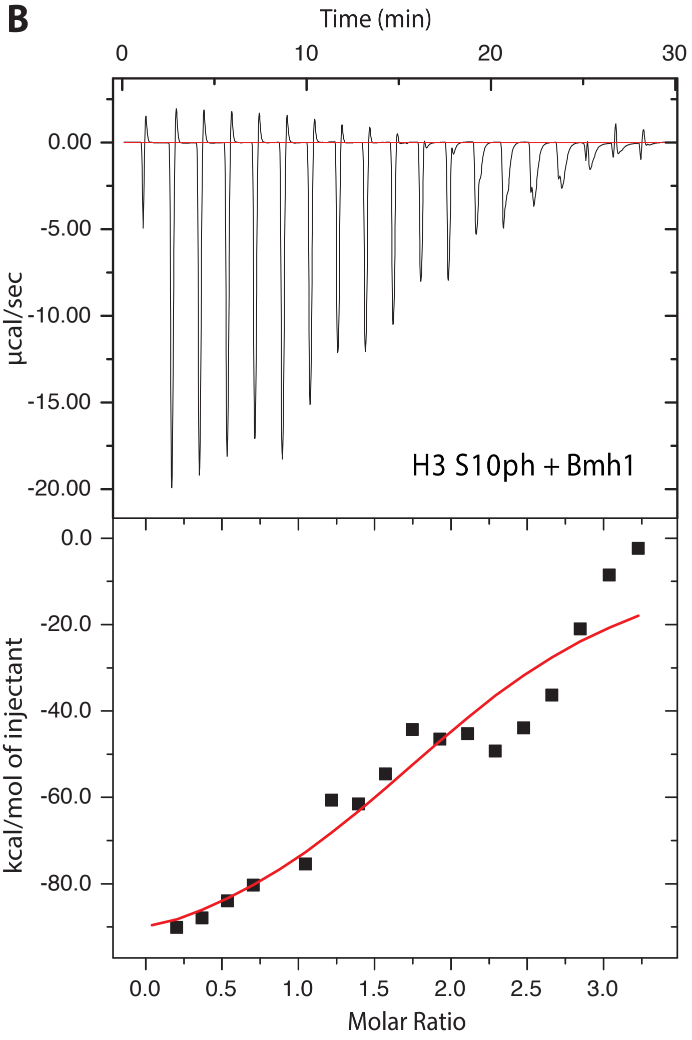

C

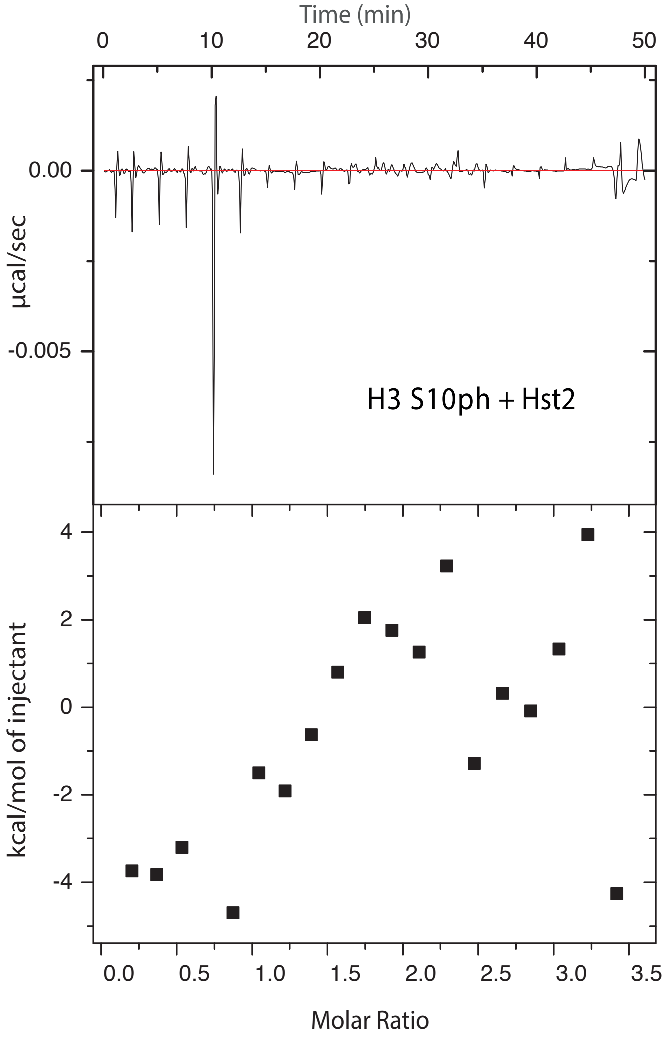

D

**Protein**	**Hst2**	**Bmh1**	**Hst2**
**Peptide**	**H4 K16ac**	**H3 S10ph**	**H3 S10ph**
**N(mol/mol)**	0.403 ± 0.015	2.09 ± 0.152	NB
**K_a(M^{-1})**	1.37E6 ± 3.4E5	5.04E4 ± 2.67E4	NB
**K_d(µM)**	0.73 ± 0.003	19.8 ± 6.9	NB
**ΔH (kcal/mol)**	−1.008E4 ± 530	−435.1 ± 53.80	NB
**ΔS (kJ/mol/K)**	−5.73	−1.37	NB

Supplementary Figure 1: Isothermal titration calorimetry (ITC). A) Thermogram and binding isotherm of H4 K16ac peptide (300 µM, injected) binding to Hst2 (30 µM in reservoir) (fit ± error of the fit). B) Same as A for H3 S10ph peptide and Bmh1. C) Same as A for H3 S10ph and Hst2. D) Derived thermodynamic parameters.

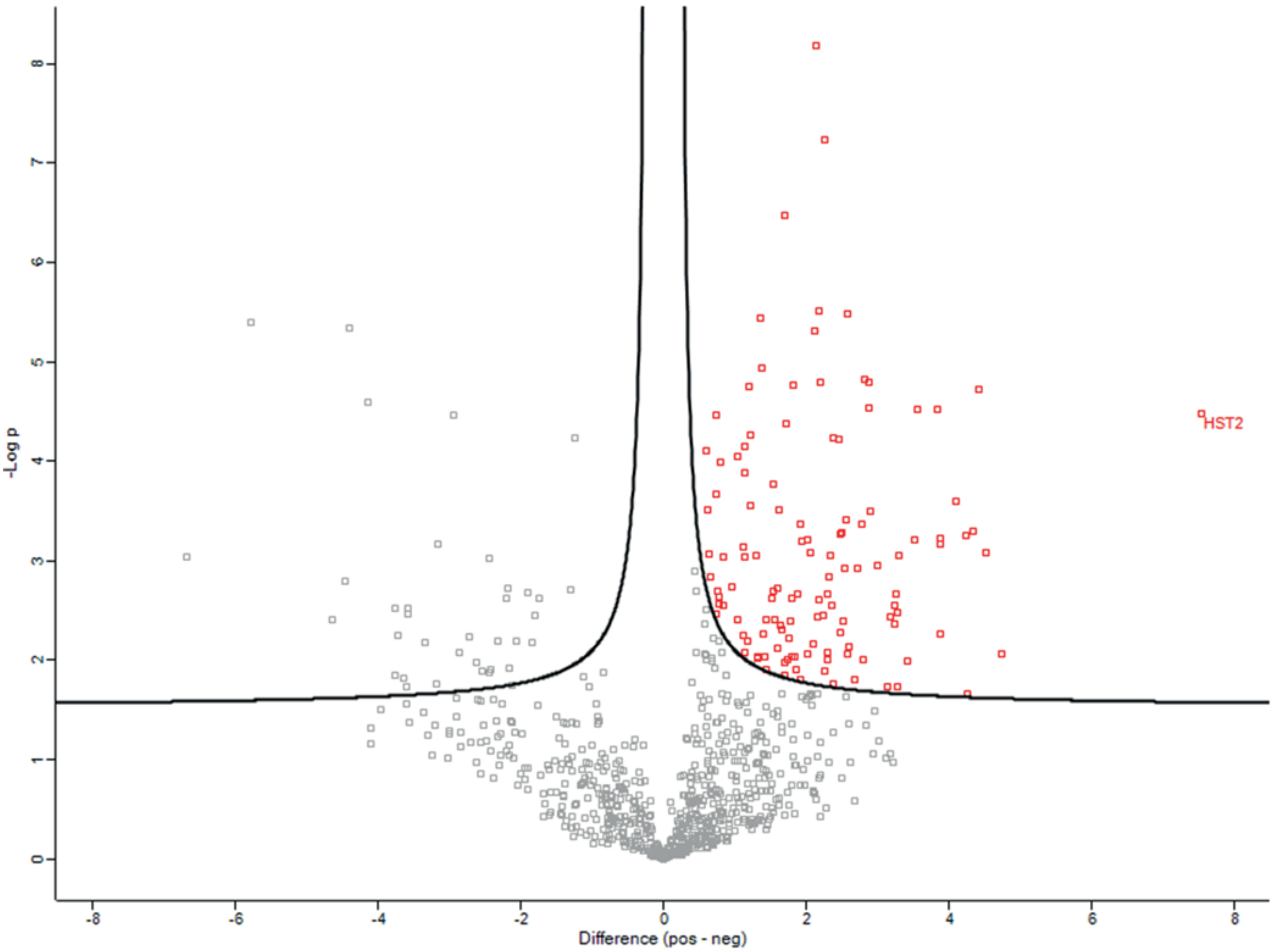

Supplementary Figure 2: Volcano Plot highlighting significantly enriched proteins in Hst2 pulldown. Analysis was performed using Perseus Software version 1.6.1.1 with a t-Test (s0=1, FDR=0.05). The statistically significant proteins with higher Log Fold change in the FLAG-tagged Hst2 (pos) sample compared to the untagged Hst2 (neg) are highlighted.

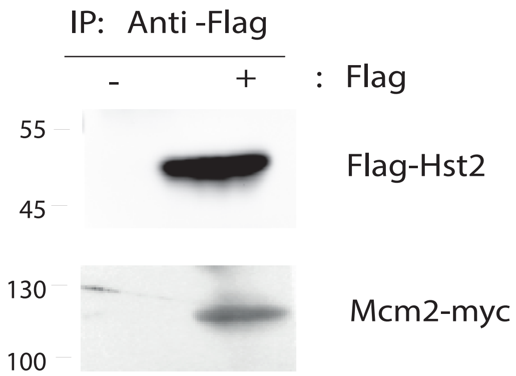

Supplementary Figure 3: Co-immunoprecipitation of Hst2 with Mcm2. Flag-IPs from lysates of yeast cells overexpressing Hst2 with or without N-terminal Flag-tag and carrying a 9xmyc-tag on Mcm2 were analyzed by SDS-PAGE and Western blot using the indicated antibodies.

EQLLEIVHDLENLSLDQS(320)EHES(324)ADKK

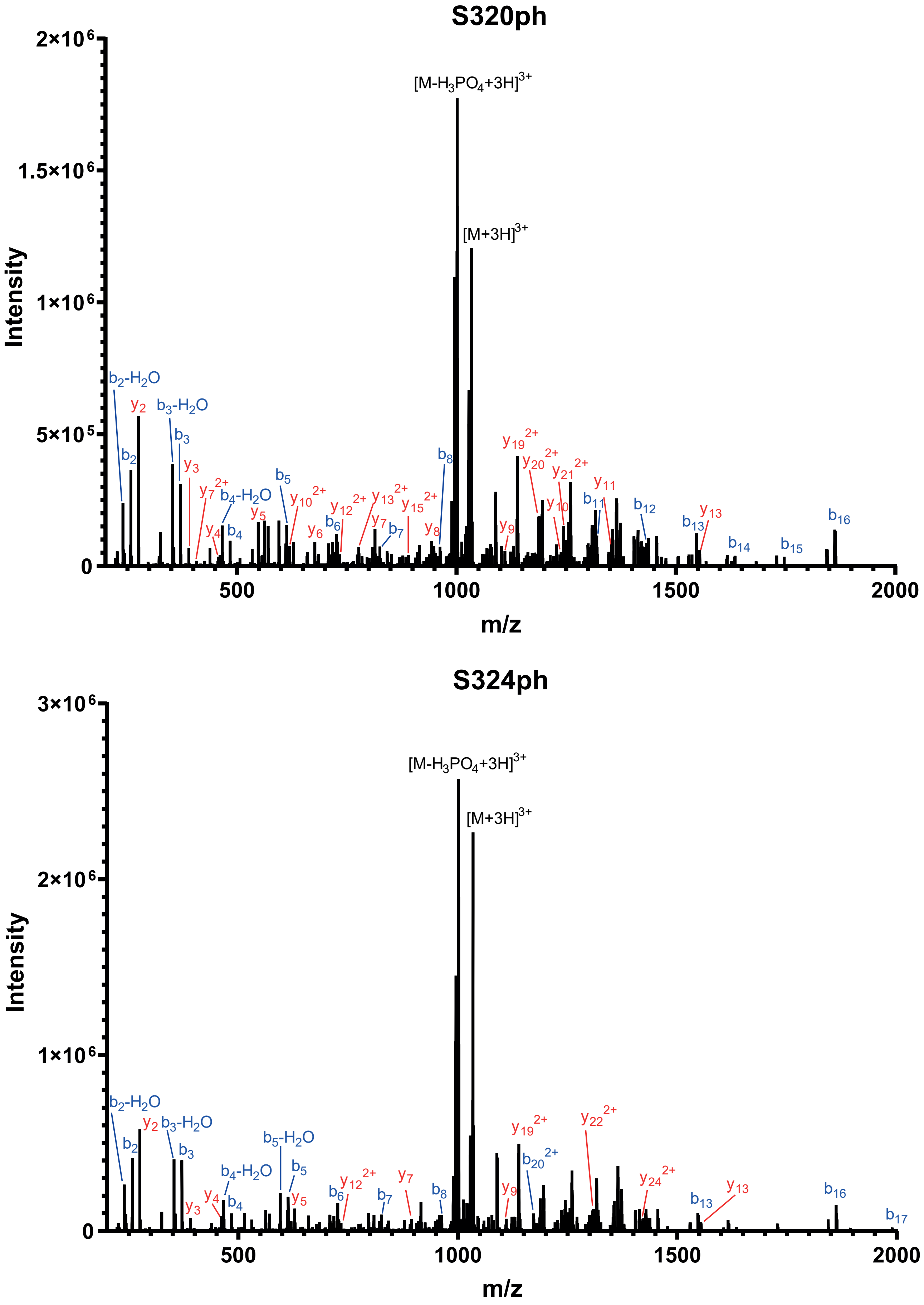

Supplementary Figure 4: MS/MS-spectra of phosphorylated Hst2-peptides.

Figure 2B:

Figure 2A:

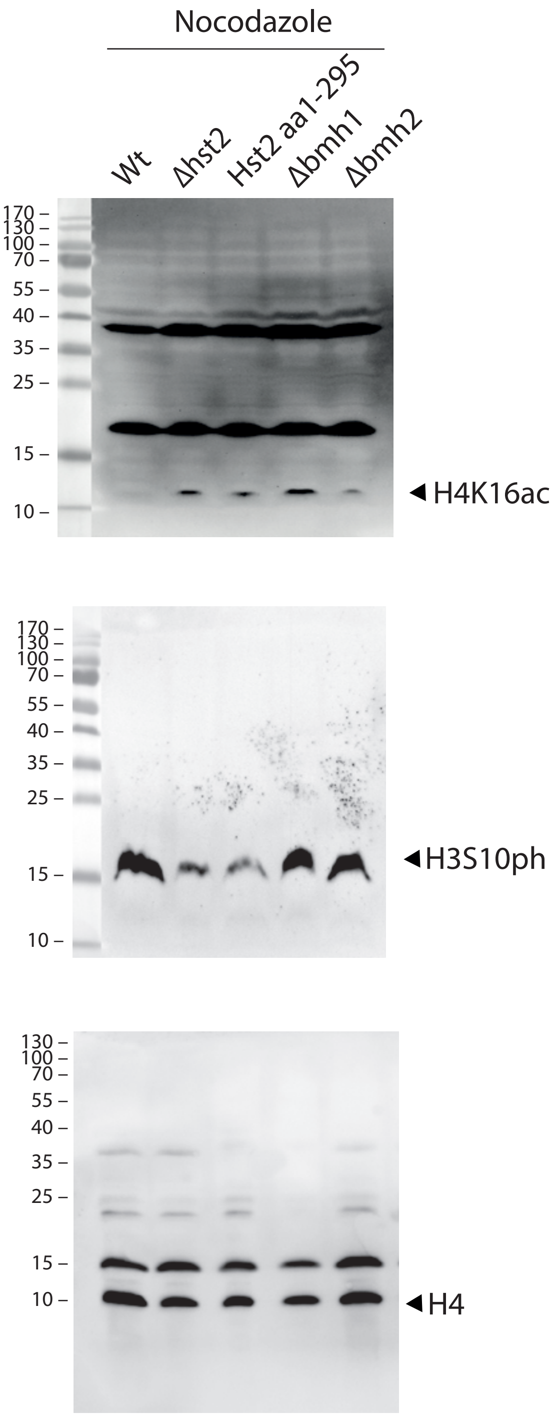

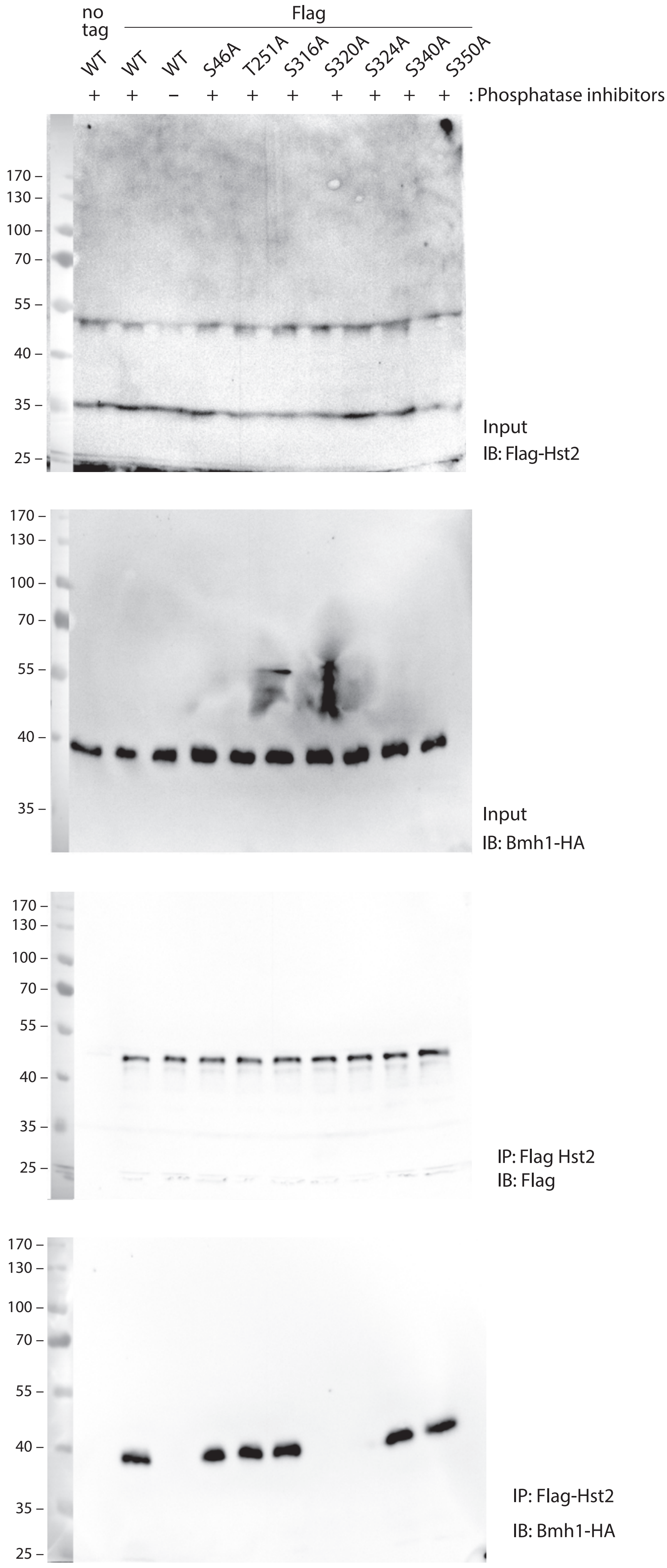

Supplementary Figure 5: Full-sized blots to Figures 2A and 2B.

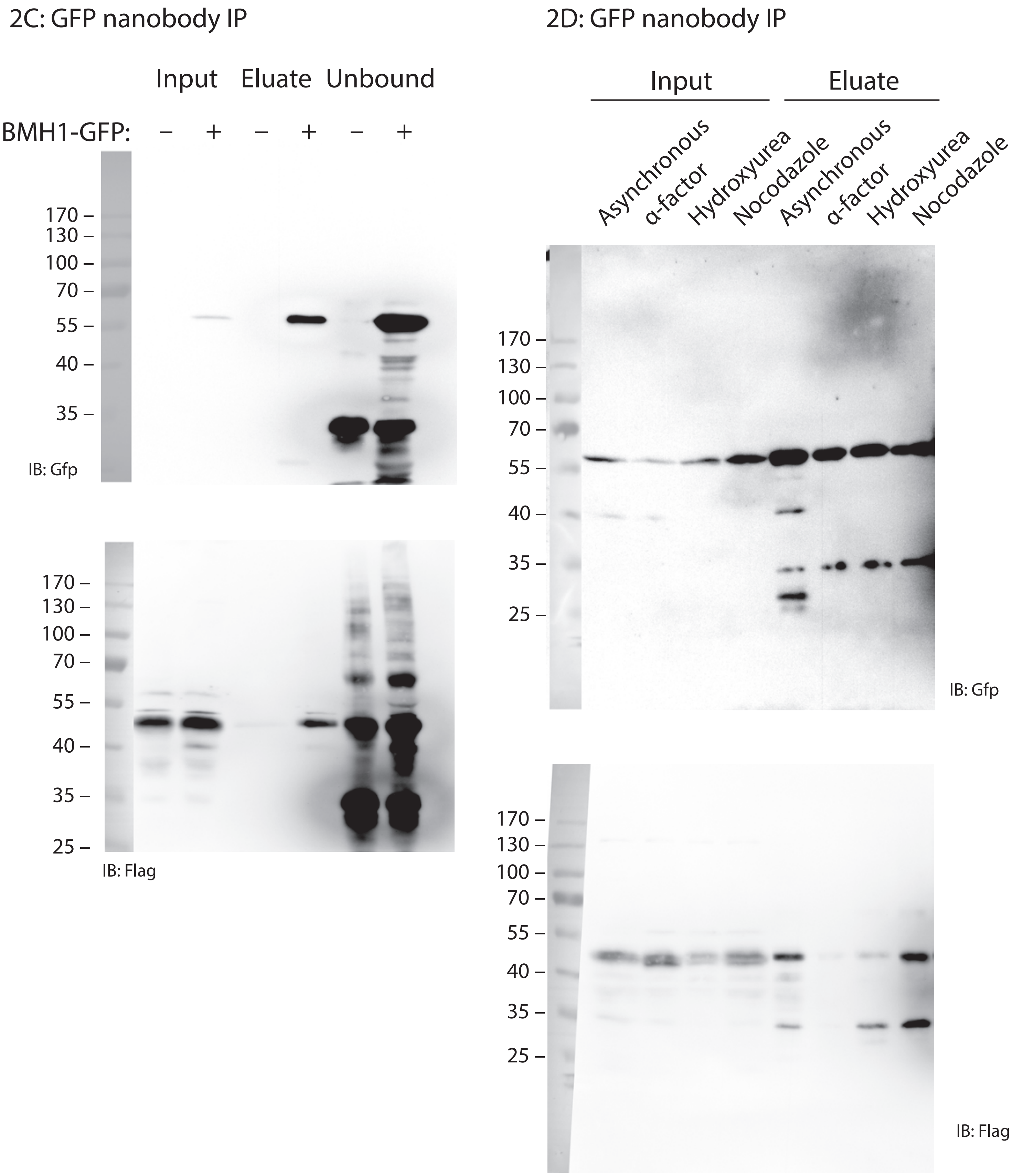

Supplementary Figure 6: Full-sized blots to Figures 2C and 2D.

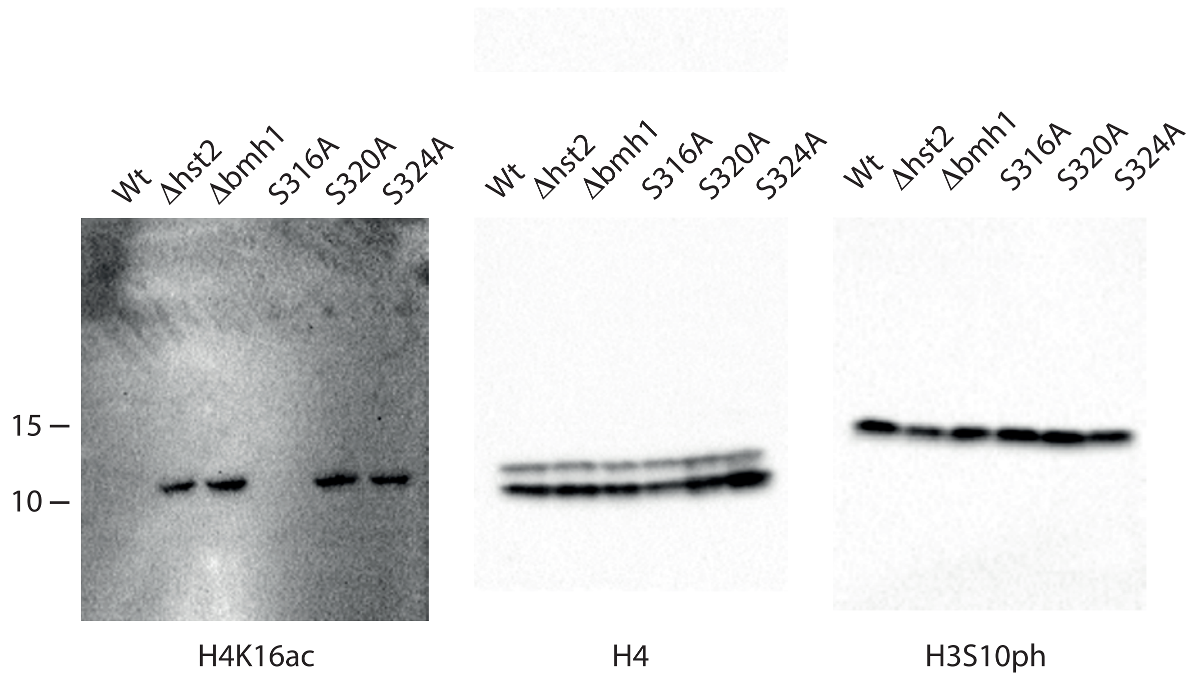

Supplementary Figure 7: Full-sized blots to Figure 2E.

SUPPLEMENTARY MATERIALS AND METHODS

Plasmids used in this study

pRS423-Hst2 and pRS423-FLAG-Hst2

The HST2-ORF with approximately 500 bp flanking DNA sequences was amplified from yeast genomic DNA and cloned SpeI/XhoI in pRS423. An N-terminal FLAG-tag was inserted by Quikchange mutagenesis.

pCDF-His-Hst2 and amber mutants thereof

The HST2 gene was first amplified from *S. cerevisiae* genomic DNA and cloned into pCDF Duet-1 using the EcoRI/PstI restriction sites. The DNA sequence encoding the N-terminal His_6-tag and linker was changed to NH_2-RGSH_6GMASM(Start)– by Quikchange mutagenesis.
Individual amber mutations were introduced by Quikchange mutagenesis at codons for S316, S320 and S324.

pCDF-His-Bmh1 and pCDF-Strep-Bmh1

The BMH1 gene was amplified from *S. cerevisiae* genomic DNA and cloned into the pCDF-Duet-1 expression vector using the BamHI/XhoI restriction sites, simultaneously introducing a TEV protease cleavage site between the His_6-tag and the N-terminus of Bmh1.
For construction of the plasmid expressing StreptII-Bmh1 in *E. coli*, Quikchange mutagenesis was used to replace the His_6-tag with a StrepII-tag to create pCDF-Strep-Bmh1.

Analysis of yeast whole cell extracts

Wild-type and mutant yeast cells were grown to OD_{600} = 0.5-1.0 and harvested equivalent to 12 OD units for each strain at 4000 rpm for 2 minutes. Cells were resuspended in 1 mL ddH_2O

(2 mM PMSF, 1 x PIC) and supplemented with 150 µL 2 M NaOH and 12 µL β-mercaptoethanol. Samples were incubated at RT for 10 minutes. Lysates were then precipitated by adding 100% TCA to a final concentration of 15% (v/v), centrifuged at 14000 rpm for 5 minutes, and washed twice with 1 mL acetone. Proteins were extracted from the pellet by boiling in SDS-PAGE sample buffer and analyzed by SDS-PAGE and Western blot.

Mass spectrometry

Sample preparation

For mass spectrometry, samples from the pull-down were digested in gel using proteomics grade trypsin protease (Sigma Aldrich). Before digestion, each gel lane was horizontally cut into small pieces (~1 mm^3 cubes) with a surgeon knife and transferred with a spatula into Protein Low binding Eppendorf tubes (Thermo Scientific Pierce) containing 500 µl of Fixation solution (5:4:1 - H_20:EtOH:Acetic acid). The tubes were incubated at 300 rpm in a Thermoshaker overnight at RT, followed by centrifugation at 14000 rpm for 10 secs next day. The supernatant was removed, the gel pieces were resuspended in 200 µl washing solution1 (3:1 – 25 mM NH_4HCO_3: Acetonitrile) and incubated for 30 min at 37°C and 600 rpm. Again, the tubes were centrifuged and the supernatant removed. The gel pieces were washed in 200 µl of washing solution 2 (1:1 – 25 mM NH_4HCO_3: Acetonitrile) and incubated for 15 min at 37°C and 600 rpm. Following this the proteins were reduced in 200 µl reducing solution (50 mM DTT in 25 mM NH_4HCO_3) for 45 min at 37°C and 600 rpm. The reducing solution was exchanged for alkylating solution (55 mM iodoacetamide in 25 mM NH_4HCO_3). The gel pieces were washed subsequently twice with washing solution 2 and once with acetonitrile. The gel pieces were dried in hood for 30 min and resuspended in 75 µl trypsin buffer, followed by an incubation for 15 min at 600 rpm. Following which, the samples were incubated at 30°C

overnight at 350 rpm in 75 µl 25 mM NH_4HCO_3. Next day, the reactions were stopped by addition of 10 µl 10% Trifluoroacetic acid and sonicated for 30 min on ice. Gel pieces were dried using 75 µl Acetonitrile. Eluted samples were dried using a Speedvac and stored at −20°C until measurement. Dried samples were then analyzed by HPLC/MS/MS. Data evaluation was performed using MaxQuant software and data visualization was done with Perseus Software version 1.6.1.1.

NanoHPLC-MS/MS

After tryptic digestion and purification, the protein fragments were analyzed by nano-HPLC-MS/MS by using an Ultimate™ 3000 RSLC nano-HPLC system, and a Q Exactive™ Plus Hybrid Quadrupole-Orbitrap mass spectrometer equipped with a nano-spray source (all from ThermoFisher Scientific). Briefly, the lyophilized tryptic peptides were suspended in 20 µL 0.1% TFA, and 3 µL of the samples were injected onto and enriched on a C18 PepMap 100 column (5 µm, 100 Å, 300 µm ID * 5 mm, Dionex, Germany) using 0.1% TFA, at a flow rate of 30 µL/min, for 5 min. Subsequently, the peptides were separated on a C18 PepMap 100 column (3 µm, 100 Å, 75 µm ID * 50 cm) using a linear gradient, starting with 95% solvent A/5% solvent B and increasing to 30.0% solvent B in 90 min, with a flow rate of 300 nL/min (solvent A: water containing 0.1% formic acid; solvent B: acetonitrile containing 0.1% formic acid). The nano-HPLC apparatus was coupled online with the mass spectrometer using a standard coated Pico Tip emitter (ID 20 µm, Tip-ID 10 µM, New Objective, Woburn, MA, USA). Signals in the mass range of m/z 300 to 1650 were acquired at a resolution of 70,000 for full scan, followed by up to ten high-energy collision-dissociation (HCD) MS/MS scans of the most intense at least doubly charged ions at a resolution of 17,500.

Protein interaction partner identification and relative quantification were performed by using MaxQuant v.1.6.1.0 (1), including the Andromeda search algorithm and searching the Saccharomyces cerevisiae reference proteome of the UniProt database. Briefly, an MS/MS ion search was performed for enzymatic trypsin cleavage, allowing two missed cleavages. Carbamidomethylation was set as a fixed protein modification, and oxidation of methionine and acetylation of the N-terminus were set as variable modifications. The mass accuracy was set to 20 ppm for the first search, and to 4.5 ppm for the second search. The false discovery rates for peptide and protein identification were set to 0.01. Only proteins for which at least two peptides were quantified were chosen for further validation. Relative quantification of proteins was performed by using the label-free quantification algorithm implemented in MaxQuant. All experiments were performed in technical duplicates of biological duplicates. Statistical data analysis was performed using Perseus v.1.6.1.1 (2). Label-free quantification (LFQ) intensities were log-transformed (log2); samples from pulldown experiments using Flag-HST2 were grouped together and samples from control experiments were also grouped together. Proteins had to be quantified at least three times in at least one of the groups to be retained for further analysis. Missing values were imputed using small normal distributed values (width 0.3, down shift 1.8), and a *t*-test (s0 = 0.01, FDR = 0.05) was performed. Proteins that were statistically significant outliers and enriched in samples from experiments using Flag-HST2 were considered as hits.

For the identification of HST2 phosphorylation sites the MaxQuant search was repeated with the same parameters, but phosphorylation of S, T, and Y were included as variable modifications.

Isothermal Calorimetry

ITC experiments were performed in 20 mM HEPES pH 7.5, 100 mM NaCl, 0.5 mM TCEP at 30°C using an iTC200 microcalorimeter (MicroCal). 300 µM peptide were titrated into the cell containing 30 µM of Hst2. The reference cell contained water. To obtain the K_d value, binding isotherms were fitted with the nonlinear least squares method, assuming one set of binding sites, by Origin (Version 7.0, MicroCal).

Peptides used: H3 S10D: NH_2-ARTKQTARKDTGGKAPRKQLK(Biotin)-COOH

H4 K16Ac: NH_2-KGGAK_{Ac}RHRKIL-COOH

FACS analysis

For the analysis of the DNA content, yeast cells were grown in (YPD) medium to an OD_{600} of approximately 0.4–0.6. 1x10^{6} cells were fixed in 70% ethanol overnight at 4°C, harvested, resuspended in 1 mL 50 mM citrate buffer, pH 7.4 and sonicated for 10 seconds at setting 30% (1 s on, 1 s off) to disperse any potential clumps of cells. Cells were harvested, resuspended in 1 mL of 50 mM citrate buffer containing 0.25 mg/mL RNAse-A (Sigma) and incubated for 2 hours at 50°C. Proteinase-K (1 mg, Sigma) was added and cells were incubated for additional 2 hours. Cells were harvested and resuspended in 1 mL 50 mM citrate buffer, pH 7.4, containing 20 µg/mL Sytox green (Sigma). Cells were incubated for 30 min at room temperature (RT), sonicated as above for 5 seconds and analyzed on a FACS BD Accuri™ C6 Plus flow cytometer (BD Biosciences), acquisition of 1x10^{4} events. The proportion of cells in each cell cycle stage was assessed with Flowjo software (version 9.0.2, Tree Star Inc, San Carlo, CA), Watson pragmatic model which fits the Gaussian DNA distribution curves to the stages of the cell cycle. Cell doublets were removed prior to analysis by plotting the area versus the width of FL-2 channel using the Flowjo software.

Phos-tag SDS-PAGE

Phospho-affinity gel electrophoresis was performed using Phos-tag^{TM} acrylamide 7.5% (w/v) separation gels polymerized with 50 µM Phos-tag^{TM} acrylamide (FUJIFILM Wako Pure Chemical Corporation) and 100 µM $ZnCl_2$. Gel running and transfer conditions were optimized according to the manufacturer's protocol.

Table S1: Yeast Strains used in this study.

Name	Strain	Genotype	Reference
Wildtype	BY4741	Mat a his3Δ1 leu2Δ0 met15Δ0 ura3Δ0	
Bmh1-GFP/Hst2-Flag	BY4741	MATa leu2D0 met15D0 ura3D0, pFA6a–GFP(S65T)–His3M Hst2-Flag:KanMX	This study
Hst2-Flag	BY4741	Mat a his3Δ1 leu2Δ0 met15Δ0 ura3Δ0 Hst2-Flag:HIS	This study
Bmh1-HA	BY4741	Mat a his3Δ1 leu2Δ0 met15Δ0 ura3Δ0 Bmh1-3HA:hphNt1	This study
Δhst2	BY4741	Mat a his3Δ1 leu2Δ0 met15Δ0 ura3Δ0 Hst2::KanMX	Yeast knockout Collections (GE DHARMACON,USA)
Δbmh1	BY4741	Mat a his3Δ1 leu2Δ0 met15Δ0 ura3Δ0 Bmh1::KanMX	Yeast knockout Collections (GE DHARMACON,USA)
Δbmh2	BY4741	Mat a his3Δ1 leu2Δ0 met15Δ0 ura3Δ0 Bmh2::KanMX	Yeast knockout Collections (GE DHARMACON,USA)
Hst2 Truncation aa295-end	BY4741	Mat a his3Δ1 leu2Δ0 met15Δ0 ura3Δ0 HST2::pRS303-Hst2_1-294	This study
Mcm2-9Myc	BY4741	Mat a his3Δ1 leu2Δ0 met15Δ0 ura3Δ0Trp::KanMX Mcm2-9Myc::KlTrp1	This study
Cdc28-Gfp	BY4741	Mat a his3Δ1 leu2Δ0 met15Δ0 ura3Δ0 Cdc28-Gfp:His	This study
Hst2 S316A	BY4741	MATa leu2D0 met15D0 ura3D0, pFA6a–GFP(S65T)–His3M Hst2S316A-Flag:KanMX	This study
Hst2 S320A	BY4741	MATa leu2D0 met15D0 ura3D0, pFA6a–GFP(S65T)–His3M Hst2S320A-Flag:KanMX	This study
Hst2 S324A	BY4741	MATa leu2D0 met15D0 ura3D0, pFA6a–GFP(S65T)–His3M Hst2S324A-Flag:KanMX	This study

Table S2: Antibodies and dilutions applied in this study

Company	Target	Host	Dilution
Primary			
Abcam (ab9110)	HA tag	Rabbit	1:10K in 3% BSA-PBS
Active Motif (39167)	Histone H4 acetyl Lys16	Rabbit	1:2.5K in 3% Milk-TBS
Cell Signaling (9701)	Phospho-Histone H3(Ser10)	Rabbit	1:3K in 3% BSA-TBS
Abcam (ab10158)	Histone H4	Rabbit	1:2.5K in 3% Milk-TBS
Abcam (ab6556)	GFP	Rabbit	1.5K in 5% Milk-TBS
Millipore (05-419)	Myc	Mouse	1.5K in 3% Milk-TBS
Secondary			
Abcam (ab6721)	Rabbit	Goat	1:10K in 3% Milk-TBS
Abcam (ab6789)	Mouse	Goat	1:10K in 3% Milk-TBS

References

1. Cox, J., and Mann, M. (2008) MaxQuant enables high peptide identification rates, individualized p.p.b.-range mass accuracies and proteome-wide protein quantification. *Nat Biotechnol* **26**, 1367-1372
2. Tyanova, S., Temu, T., Sinitcyn, P., Carlson, A., Hein, M. Y., Geiger, T., Mann, M., and Cox, J. (2016) The Perseus computational platform for comprehensive analysis of (prote)omics data. *Nat Methods* **13**, 731-740
